# Ionosphere-Constrained Single-Frequency PPP with an Android Smartphone and Assessment of GNSS Observations

**DOI:** 10.3390/s20205917

**Published:** 2020-10-20

**Authors:** Guangxing Wang, Yadong Bo, Qiang Yu, Min Li, Zhihao Yin, Yu Chen

**Affiliations:** 1 School of Geography and Information Engineering, China University of Geosciences, Wuhan 430078, China; wanggx@cug.edu.cn (G.W.); Boyadong@cug.edu.cn (Y.B.); yuqiang@cug.edu.cn (Q.Y.); yinzhihao@cug.edu.cn (Z.Y.); 2 GNSS Research Center, Wuhan University, Wuhan 430079, China; limin@whu.edu.cn

**Keywords:** Android smartphones, GNSS raw observation, Precise Point Positioning (PPP), multipath, BDS

## Abstract

With the development of Global Navigation Satellite System (GNSS) and the opening of Application Programming Interface (API) of Android terminals, the positioning research of Android terminals has attracted the attention of GNSS community. In this paper, three static experiments were conducted to analyze the raw GNSS observations quality and positioning performances of the smartphones. For the two experimental smartphones, the numbers of visible satellites with dual-frequency signals are unstable and not enough for dual-frequency Precise Point Positioning (PPP) processing all through the day. Therefore, the ionosphere-constrained single-frequency PPP model was employed to improve the positioning with the smartphones, and its performance was evaluated and compared with those of the Single Point Positioning (SPP) and the traditional PPP models. The results show that horizontal positioning accuracies of the smartphones with the improved PPP model are better than 1 m, while those with the SPP and the traditional PPP models are about 2 m.

## 1. Introduction

With the development of Global Navigation Satellite System (GNSS) in the 21th century, the number of available navigation satellites has increased significantly. It has formed a multi-system global navigation constellation framework mainly composed of American Global Positioning System (GPS), Russian Global Navigation Satellite System (GLONASS), Chinese BeiDou Navigation Satellite System (BDS) and EU’s Galileo system [1]. Moreover, Japanese Quasi-Zenith Satellite System (QZSS) is widely used to supplement GPS services as a regional navigation satellite system. The development of GNSS also promotes the continuous development of location-based service business based on smartphones, and greatly facilitates both industrial production and daily life.

For a long time, only rough positioning results could be obtained from the smartphones. Launched in 2016, the Android 7.0 operating system supports the access to GNSS raw measurements and navigation messages [2], making the Android devices function more like a GNSS receiver. In the same year, Google released GNSSLogger, an open-source program that could help retrieve GNSS raw observations from Android smartphones, including the observations of code pseudorange, carrier phase, and Doppler [3]. In 2017, Geo++ released an application named Geo++ RINEX Logger to provide GNSS raw observation directly in RINEX format [4]. Both of the above two applications cannot output the information of navigation messages. RinexON, released by Flamingo team at the end of June 2018, is able to output multi-GNSS raw data, including broadcast ephemeris, collected by smartphones in RINEX 3.0.3 format [5]. The accessibility of GNSS raw data makes it possible to analyze the observation quality and to study the positioning algorithm with Android terminals.

In recent years, GNSS positioning with Android terminals has become one of research focuses. The experiment by Gim et al. [6] is a Single Point Positioning (SPP) test with code measurements of the Nexus 9 tablet, and the results show that the RMS of positioning errors in horizontal and three-dimensional (3D) are 3.05 m and 3.82 m. In an experiment of double-differenced positioning with single-frequency carrier phases from the tablet and several base stations, the positioning accuracy better than 20 cm can be achieved within 20 min [7]. The carrier-to-noise ratio (C/N0) value of GNSS raw observations collected by the Nexus 9 tablet is 10 dBHz lower than the representative values obtained from a geodetic-quality antenna and receiver. With time-differenced filtering method, horizontal and vertical accuracies of static positioning can be better than 0.6 and 1.4 m, respectively [8]. Martin [9] also used a Nexus 9 tablet for positioning test. The research shows that multipath plays an important role for the expected accuracy of the calculated precise positions, both due to the induced error on the measurements, and due to loss of lock of the GNSS signals, which significantly affects precise positioning from carrier phase measurements. Although these studies have important implications for subsequent experiments using smartphones, the positioning of ordinary smartphones is not comparable to this tablet.

Many scholars have carried out differential GNSS researches on smartphones. Zhang et al. [10] developed an Android application based on wide area differential location technology, and the static observation results show that the horizontal accuracy of the smartphones can reach about 4 m. The maritime test by Specht et al. [11] showed that the accuracy of the dynamic positioning with smartphones during vessel maneuvering can reach 10 m, satisfying most of the maritime requirements for navigation accuracy. The Network Real Time Kinematic (NRTK) positioning accuracy with smartphones by Dabove et al. [12] is about 60 cm. Moreover, Wanninger et al. [13] performed carrier phase ambiguity fixing for smartphones. With ambiguities successfully fixed, the 3D positioning accuracies (standard deviations) better than 4 cm could be achieved after five minutes of static observation session, and an accuracy of 2 cm is possible for long observation sessions.

Since the launch of Xiaomi 8 in June 2018 [14], smartphones supporting dual-frequency GPS signals have become the mainstream of the market and motivate the research of smartphones Precise Point Positioning (PPP). The quality analysis results of GNSS raw observations show that the number of visible satellites with Xiaomi 8 is similar to that with geodetic receivers, although the carrier-to-noise ratio and multipath effect with smartphones are worse than the typical values with geodetic receivers [15]. It is found that carrier phase measurements collected by smartphones might contain gross errors and systematic errors, and that different clocks are used for code and carrier phase observations [16]. By considering the clock bias between the code and carrier phase measurements, the accuracy of PPP can be better than 1 m [17]. Shi et al. [18] conducted static and dynamic observation experiments with Samsung S8, Huawei Mate20 and Xiaomi8. After experiments, he evaluated the GNSS data quality of smartphones in detail. Through proper GNSS data quality control, he initially achieved positioning accuracy within 1 m. Wu et al. [19] conducted long-term static observation with smartphones. The PPP results show that the positioning accuracy of smartphone with dual frequency data is better than 20 cm, but it takes up to 100 min to converge. These studies confirm the practicability of using GNSS raw data of smartphones to realize PPP, which is of great significance for subsequent PPP studies using smartphones.

According to the above published researches, the difficulties of precise positioning with smartphones were found. Compared with geodetic receivers, smartphones are prone to suffer from frequent losses of lock, unstable clock and poor quality of measurements, due to the relatively low-cost GNSS chips and antennas [15,16,17,18]. Therefore, the GNSS raw observations collected by smartphones are likely to contain a large number of cycle slips, and to be seriously affected by multipath effects and low carrier-to-noise ratio, jeopardizing the PPP accuracies with smartphones. To overcome the disadvantages of smartphones in GNSS data collection, it is necessary to quantitatively analyze the quality of observations of smartphones and to study the method of cycle slip detection.

Although some types of smartphones are designed with the nominal capabilities of tracking dual-frequency GNSS signals, the real measurements are usually far from enough for dual-frequency PPP processing all through the day with the traditional method, due to various reasons such as the frequent interruptions of dual-frequency data. Thus, the single-frequency PPP is still a common processing mode for smartphones, and the ionospheric delay are corrected by the Global Ionospheric Maps (GIM), the products of which can correct about 80% of the ionospheric delay [20]. Although the remaining ionospheric delay can still reach decimeter level, it is sufficient for the single-frequency PPP with smartphones, considering that the precision of code pseudorange measurements from smartphones is nearly 10 m [18]. In theory, GIM can be used to build constraint equations, to improve the reliability of single-frequency PPP processing of smartphones and shorten the convergence time [21]. However, there is a lack of research on the impact of ionosphere constraints on the smartphones PPP.

This paper contributes to GNSS data quality analysis and PPP with Android smartphones. Section 2 represents the principles and methodologies of single-frequency PPP, smoothing code pseudorange and cycle slip detection, in special consideration of the characteristics of data collected by smartphones. In Section 3, the quality of GNSS measurements from two different Android devices and one geodetic receiver are analyzed and compared from the aspects of satellite tracking performance, carrier-to-noise ratio and multipath effects, followed by PPP experiment with GNSS Analysis Software for Multi-constellation and Multi-frequency Precise Positioning (GAMP) [22]. Conclusions are given in Section 5.

## 2. The Principles and Methodologies of PPP with Smartphones

**Figure 1:**
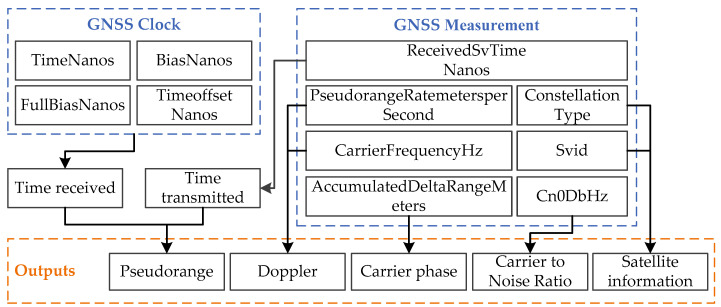
Generation principle of GNSS raw observations.

Android 7.0 provides positioning related API. The modules related to the raw GNSS observations are GNSS Clock, GNSS Measurement and GNSS Navigation Message. The module GNSS Clock provides quartz clock information of the Android smartphones. The module GNSS Measurement provides observation information of each satellite signal obtained by base frequency processing. The module GNSS Navigation Message provides satellite ephemeris information. The modules GNSS Clock and GNSS Measurement can generate the raw GNSS observations of each satellite, including code pseudorange, carrier phase, Doppler and carrier-to-noise ratio. The generation principle of GNSS raw observations through the modules GNSS Clock and GNSS Measurement is shown in Figure 1 [18].

### 2.1. Ionosphere-Constrained Single-Frequency PPP

Since the numbers of dual-frequency observables tracked by the current smartphones are far from sufficient, the single-frequency PPP model is still the main choice for the positioning research with smartphones at present [17]. Without dual-frequency observables, the ionospheric delay is unable to be reduced through the ionosphere-free combination, and has to be estimated as an additional unknown parameter. Besides, the qualities of code and carrier phase measurements received by smartphone are usually not as high as those by geodetic receiver [18]. To improve the PPP accuracy with smartphone, external ionospheric information is introduced as virtual observations. The model of single-frequency PPP with ionospheric constraint for one satellite-receiver pair can be expressed as.

(1) $$\left[\begin{array}{c} p\\ \begin{array}{c} \varphi \\ \tilde{I} \end{array} \end{array}\right]=\left[\begin{array}{cc} \begin{array}{cc} \begin{array}{c} u\\ u\\ 0 \end{array} & \begin{array}{c} 1\\ 1\\ 0 \end{array} \end{array} & \begin{array}{ccc} \begin{array}{c} M\\ M\\ 0 \end{array} & \begin{array}{c} 1\\ -1\\ 1 \end{array} & \begin{array}{c} 0\\ \lambda \\ 0 \end{array} \end{array} \end{array}\right]\left[\begin{array}{c} x\\ \delta t\\ \begin{array}{c} Z_{w}\\ I\\ N \end{array} \end{array}\right]+\left[\begin{array}{c} \varepsilon \\ \xi \\ \zeta \end{array}\right],Q_{p},Q_{\varphi },Q_{I}$$

where p and φ represent the observed-minus-computed (OMC) values of the code pseudorange and the carrier phase, respectively; $\tilde{I}$ denotes the virtual observable of the ionospheric constraint calculated by the GIM products; u is the line-of-sight direction vector specific to each satellite; x is the incremental vector of the receiver position with respect to the initial approximate coordinates; δt and $Z_{w}$ are the parameters of receiver clock and Zenith Wet Delay(ZWD) specific to the station; M and λ are the mapping function of ZWD and the signal wavelength; I and  N are the ionospheric delay and carrier phase ambiguity specific to certain station, satellite and frequency; ε, ξ and ζ are the sums of unmodeled errors and observing noise corresponding to each observable; $Q_{p}$, $Q_{\varphi }$ and $Q_{I}$ represent the variances of the corresponding observables. It is worth noting that all the indices of satellite, receiver and frequency have been omitted for simplicity.

In this study, the rapid products of orbits and clocks released by the Multi-GNSS Experiment (MGEX) are adopted, and the satellite Differential Code Biases (DCBs) are corrected by the DCB products from the Center for Orbit Determination in Europe (CODE) and the MGEX. The satellite antenna phase center offsets and variations, relativistic effects, zenith dry delay, tidal loadings, and phase windup are corrected by the empirical models [23].

### 2.2. Smoothing Code Pseudorange with Doppler

Although the method of phase smoothing pseudorange is usually used to reduce the noise of code measurements, its performance is prone to be affected by the continuity of phase observation, especially in the cases of poor tracking conditions with smartphones. Compared with the phase observation, the Doppler observation is unlikely to be affected by cycle slips, and its accuracy is higher than that of the code measurement. Therefore, the Doppler observation can be used alternatively to reduce the noise and multipath error of the code measurement [24].

The integral Doppler is equal to the variation of carrier phase during the integral interval, which reflects the variation of geometric distance between the satellite and the smartphone. The algorithm of Doppler smoothing code pseudorange can be expressed as

(2) $${\bar{P}}_{t_{i}}=\frac{1}{m}P_{t_{i}}+\frac{m-1}{m}\left({\bar{P}}_{t_{i-1}}+\lambda \cdot \int_{t_{i-1}}^{t_{i}}Ddt\right)$$

where $t_{i}$ and $t_{i-1}$ are two adjacent moments; P and $\bar{P}$ represent the raw and smoothing code pseudorange; D is the Doppler observation; m is a constant set as 75 in the study.

### 2.3. Cycle Slip Detection

Due to the lack of dual-frequency GNSS observations, cycle slip detection is another challenging task for the smartphones. At present, the main cycle slip methods for single-frequency scenario include code-phase comparison, Doppler integration, and higher-order time differencing of carrier phase observations [25,26,27]. In this study, the methods of code-phase comparison and Doppler integration are employed for the detection of cycle slips contained in the data collected by the smartphones.

In the observation equation, the terms of geometric distance, receiver clock and satellite clock can be eliminated through the differencing between the raw measurements of the code pseudorange P and the carrier phase Φ, so we have

(3) Δ=P−Φ=2I−λN+ε−ξ

where the parameters have the same meaning as Equation (1).

If no cycle slip occurs, the ambiguity parameter stays constant. The ionospheric delay varies slowly, and so is the code-phase differencing Δ. The temporal variation of Δ stays relatively stable unless there are cycle slips. As a consequence, the between-epoch differencing of Equation (3) can be used to detect and determine the possible cycle slips.

**Figure 2:**
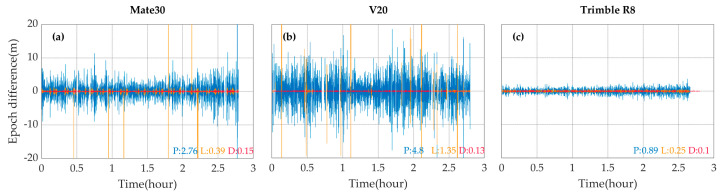
The third-order between-epoch differences of code (blue) and carrier phase (orange) measurements, as well as the second-order between-epoch differences of the Doppler (red) measurements on L1 frequency of G26 satellite with the Mate30 (**a**), the V20 (**b**) and the Trimble R8 (**c**). The numbers with different colors listed at the bottom right corner of each panel represent the corresponding standard deviations.

The abovementioned cycle slip detection method is highly dependent on the quality of the code measurements. To obtain the knowledge about the precisions and stabilities of the measurements with different devices, we investigate the high-order between-epoch differences of raw measurements, and shown in Figure 2. are the typical third-order between-epoch differences of the code (blue) and the carrier phase (orange) measurements, as well as the second-order between-epoch differences of the Doppler (red) measurements.

The code measurements of the two smartphones are far less precise than the those of the geodetic receiver, which is likely to undermine the performance of the cycle slip detection method based on Equation (3). However, the Doppler measurements of all the three devices are comparable in precision and stability, suggesting that the Doppler measurements can be used as supplements of the code and phase measurements in the detection of cycle slips.

Therefore, an indicator based on the difference between the carrier phase and Doppler integral are calculated as [27]

(4) $$\delta N=\frac{\Delta \Phi \left(t_{i-1},t_{i}\right)-\int_{t_{i-1}}^{t_{i}}Ddt}{\lambda }$$

where $\Delta \Phi \left(t_{i},t_{i-1}\right)$ is the variation of the carrier phase between two adjacent epochs. An absolute value of δN larger than the threshold indicates the cycle slip.

**Figure 3:**
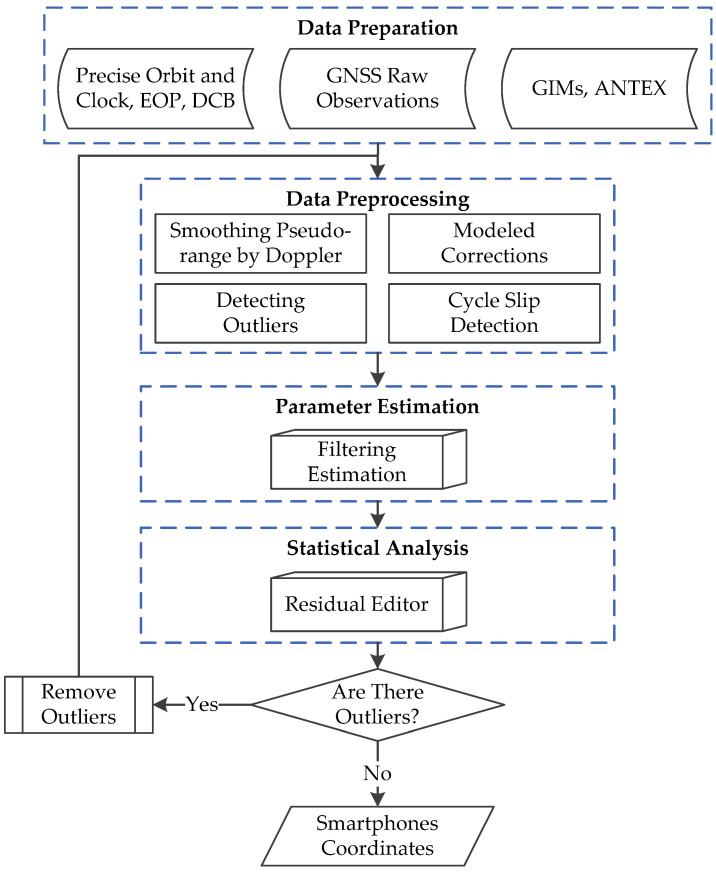
Flowchart of ionosphere-constrained single-frequency PPP processing for the smartphones.

The methods of code-phase comparison based on Equation (3) and Doppler integration based on Equation (4) are used together to guarantee the performance of cycle slip detection. In summary, the flowchart of ionosphere-constrained single-frequency PPP for smartphone is shown as Figure 3.

## 3. GNSS Data Quality Analysis

The main device used in this experiment is a Huawei Mate30 smartphone (hereinafter referred to as Mate30). For comparison analysis, a Huawei honorV20 smartphone (hereinafter referred to as V20) and a geodetic receiver (Trimble R8) were also used. The Mate30 smartphone is a dual-frequency GNSS smartphone that collects the first frequency signals of GPS, GLONASS, BDS, Galileo and QZSS, and the second frequency signals of GPS, Galileo and QZSS. Although the V20 smartphone is cheaper than the Mate30 smartphone, it also supports dual-frequency observations and all of the five systems. Listed in Table 1 are the GNSS related characteristics of the three devices used in this paper.

In the experiment, Trimble R8 is used as the reference, and its antenna is only about 10 cm away from the two smartphones. To compare the observing positioning performances of the two smartphones with those of the geodetic receiver, synchronous observations were conducted with the three devices. Data was collected on the evening of 13 November, the afternoon and the evening of 18 November 2019, and each of the three observing periods lasted for 2–3 h. Since the devices were equipped at almost the same place during the three observing periods, the difference of the surroundings could be neglected.

### 3.1. Satellite Tracking

**Figure 4:**
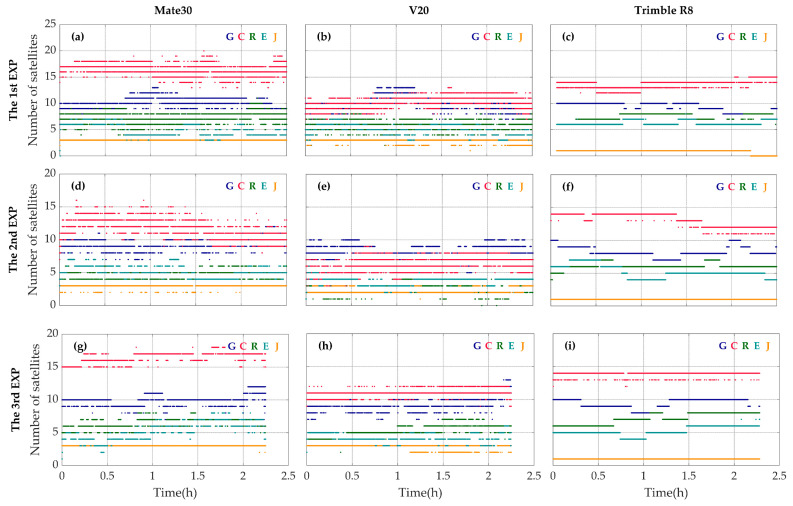
The numbers of GPS (blue), BDS (red), GLONASS (green), Galileo (cyan) and QZSS (orange) satellites with the signal of the first frequency tracked by the Mate30 smartphone (**a**,**d**,**g**), the V20 smartphone (**b**,**e**,**h**) and the geodetic receiver Trimble R8 (**c**,**f**,**i**) during the first (**a**–**c**), the second (**d**–**f**) and the third (**g**–**i**) observing periods.

Shown in Figure 4 are the numbers of different GNSS satellites with the signal of the first frequency tracked by the geodetic receiver and the two smartphones. The Mate30 smartphone is able to track about 38 satellites in total, while the V20 smartphone tracks only 30 ones. Since the GNSS chip of the Mate30 is nominally supporting BDS-3 signals, it can track more BDS satellites than the V20, which can only track BDS-2 satellites. Compared with the geodetic receiver, both of the two smartphones suffer from frequent fluctuations in the numbers of visible satellites, although they can track as many satellites as, if not more than, the geodetic receivers.

**Figure 5:**
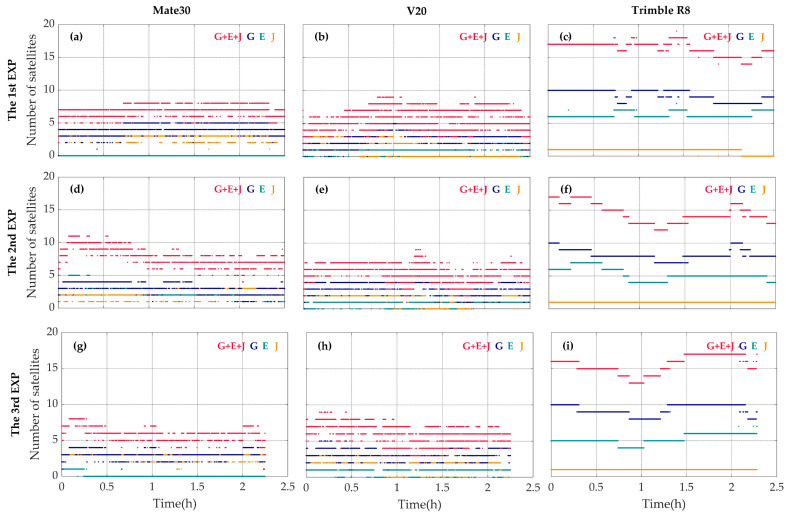
The numbers of GPS (blue), Galileo (cyan), QZSS (orange) and the total satellites of these three systems (red) with dual-frequency measurements available for the Mate30 smartphone (**a**,**d**,**g**) and the V20 smartphone (**b**,**e**,**h**) and the geodetic receiver Trimble R8 (**c**,**f**,**i**) during the first (**a**–**c**), the second (**d**–**f**) and the third (**g**–**i**) observing periods.

Shown in Figure 5 are the numbers of satellites with dual-frequency signals tracked by the Mate30, the V20 and the Trimble R8. The number of satellites with dual-frequency signals tracked by the Mate30 smartphone fluctuates between 3 and 10, while the number of satellites with dual-frequency signals tracked by the V20 smartphone fluctuates between 1 and 9. The dual-frequency measurements collected by either the Mate30 or the V20 are far from sufficient for continuous PPP experiment with the ionosphere-free combination of L1/L5 or E1/E5A observables, and the case might be worse in real scenarios with urban canyons.

The data continuities of GPS, Galileo and QZSS are also compared and the results are shown in Figure 5. The number of GPS, Galileo and QZSS dual-frequency satellites of two smartphones fluctuates seriously. And in a certain period of time, the dual-frequency satellite of Galileo cannot be observed by the two smartphones. Considering that QZSS system is a regional navigation satellite amplification system developed by Japan, adopting the Inclined Geosynchronous Orbit (IGSO), the precision of positioning service provided is limited [22]. Therefore, the reliable dual-frequency measurements collected by the smartphones are still mainly from GPS satellites at present.

### 3.2. Carrier-to-Noise Ratio

The carrier-to-noise ratio refers to the ratio of the average power of the carrier signal received at the receiver end to the average power of the noise when the signal is interfered in the process of propagation. The carrier-to-noise ratio reflects the noise level of the measurement [28]. The higher the carrier-to-noise ratio is, the better the observation quality is.

**Figure 6:**
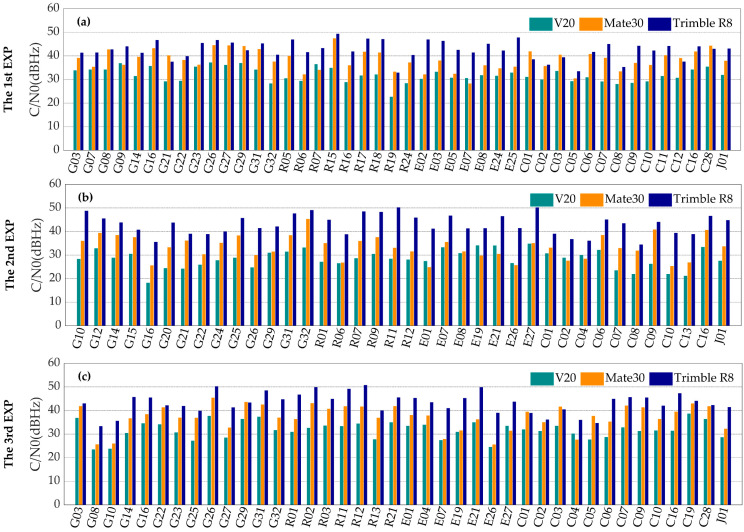
The mean carrier-to-noise ratios of the first frequency with the V20 smartphone (cyan), the Mate30 smartphone (orange) and the geodetic receiver Trimble R8 (blue) in the first (**a**), the second (**b**) and the third (**c**) experiments.

Shown in Figure 6 are the mean values of carrier-to-noise ratio in three experiments. Generally, the mean carrier-to-noise ratio with the geodetic receiver is the highest among all of the three devices, although for several BDS satellites the mean carrier-to-noise ratio with the Mate30 smartphone is the highest. The carrier-to-noise ratios with the Mate30 smartphone are around 40 dBHz, obviously better than those with the V20 smartphone. This may be due to better antenna and GNSS chip of the Mate30 smartphone. 

**Figure 7:**
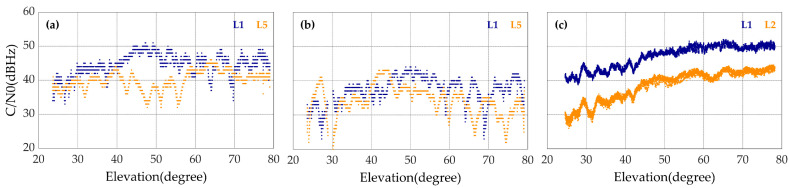
The carrier-to-noise ratios of L1 (blue), L5 or L2 (orange) code measurements of G26 satellite with the Mate30 (**a**), the V20 (**b**) and the Trimble R8 (**c**) with respect to the satellite elevation in the first experiment.

Figure 7 shows the typical relationships between the carrier-to-noise ratio and the elevation angle for the three devices. The carrier-to-noise ratio with the geodetic receiver increases as the elevation angle increases, while for the two smartphones, the correlation between carrier-to-noise ratio and elevation angle is not obvious. This could be explained by the different polarization modes. Instead of the right-handed circular polarization, the linear polarization is adopted by the built-in GNSS antennas of the smartphones. Therefore, the smartphones are more vulnerable to signal interferences.

In addition, through the comparison between the carrier-to-noise ratios of L1 and L5 observations collected by the smartphones, it is found that the carrier-to-noise ratios of the L5 observations are equivalent to or even better than those of the L1 observations in the cases of low elevations. It can also be inferred that theL5 signal outperforms the L2 signal in anti-jamming in the cases of low elevations. However, the average carrier-to-noise ratios of the L5 signals received by the smartphones are around 4 dBHz lower than those of the L1 signals. This might be due to the imperfect multi-frequency antenna design of the smartphones, and further study is needed.

### 3.3. Multipath Effect

The propagation direction, amplitude and phase of GNSS signal are prone to be affected by the reflections of the surrounding at the antenna, and the reflected signals can cause multipath effects [29]. The observing environments for smartphone users are complicated, and it is inconvenient for the antenna of a smartphone to suppress the multipath error through hardware like the choke ring. Therefore, the multipath error usually plays a dominant role in the code measurement collected by smartphone [9]. Since the multipath error of code measurement is as 200 times large as that of carrier phase [30,31], we focus on the multipath error of the code measurement in the study.

When dual-frequency observations are available, the code multipath errors can be studied with the multipath combination, which can be expressed as

(5) $$MP_{i}=P_{j}-\frac{f_{i}^{2}+f_{j}^{2}}{f_{i}^{2}-f_{j}^{2}}\Phi_{i}+\frac{2f_{j}^{2}}{f_{i}^{2}-f_{j}^{2}}\Phi_{j}$$

where the subscripts i and j (i≠j) denote different frequency bands, and f is the frequency value. The combination expressed by Equation (5) mainly contains the multipath error of corresponding code measurement and the linear combination of the ambiguities. If no cycle slip occurs, the ambiguities are considered constants and can be removed through averaging over epochs [32]. The subscripts for receiver and satellite have been omitted here for simplicity.

**Figure 8:**
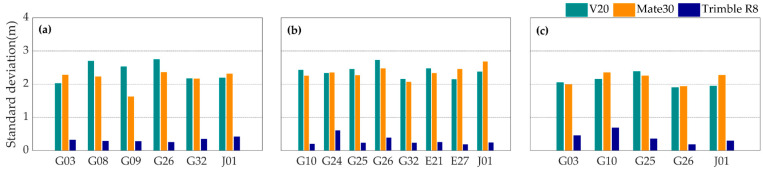
The standard deviations values of L1 frequency code multipath error of the V20 (cyan), the Mate30 (orange) and the Trimble R8 (blue) during the first (**a**), the second (**b**) and the third (**c**) observing periods.

Shown in Figure 8 are the standard deviations of L1 frequency code multipath error of the three devices in the three experiments. The standard deviations of code multipath errors of the Mate30 and the V20 are as ten times large as that of the Trimble R8. This phenomenon shows that the two smartphones have disadvantages in suppressing the multipath effects.

**Figure 9:**
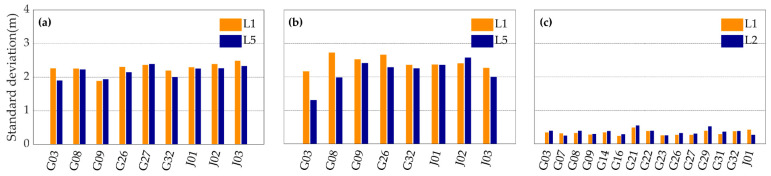
The standard deviations of L1 (orange), L5 or L2 (blue) code multipath errors with the Mate30 (**a**), the V20 (**b**) and the Trimble R8 (**c**) in the first experiment.

**Figure 10:**
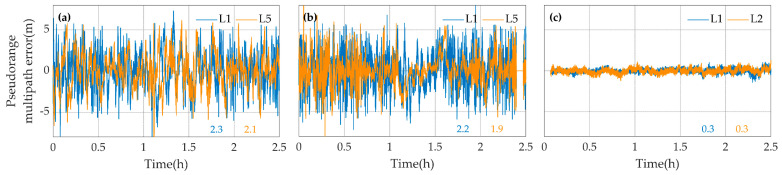
The multipath errors of L1 (blue), L5 or L2 (orange) code measurements of G26 satellite with the Mate30 (**a**), the V20 (**b**) and the Trimble R8 (**c**) in the first experiment. The standard deviations of the code multipath errors are shown with the corresponding colors in meters.

Figure 9 and Figure 10 show the typical standard deviations values and the multipath errors time series of L1, L5 or L2 code measurements with the three devices. It can be seen that the code multipath errors of the two smartphones vary from −4 and 4 m, and that the code measurements with the geodetic receiver are less affected by the multipath error. It suggests that the multipath error should be one of the main factors limiting the accuracy of PPP with the smartphones. Meanwhile, the standard deviationsof the code multipath errors of L1 and L5 code measurements are about 2 m for smartphones, and L5 code measurements are generally less affected by multipath errors than L1 code measurements.

## 4. PPP Results

In this paper, a single-frequency PPP processing strategy based on ionosphere constraints is employed. Shown in Table 2 are the setting details of the single-frequency PPP for smartphones. The products of satellite orbit, satellite clock and Earth rotation are downloaded from MGEX data center (http://www.cddis.gsfc.nasa.gov/). Ionospheric delay products are downloaded from CODE. And ionospheric delay is constrained by GIM products of CODE.

In order to compare the positioning results with the different strategies and to validate the abovementioned method, three experiments were conducted with different settings. The details of these projects are shown in Table 3. 

The standard deviations of positioning errors of the Mate30 using different projects are shown in Table 4. Considering that the horizontal position of smartphones is more widely used [10,11,12,13], the horizontal accuracy of smartphones was only recorded and analyzed. The standard deviations of positioning errors in the East and North direction of the Mate30 using the SPP and traditional PPP projects are about 2–4 m. And that of the improved PPP project is less than 1 m. Table 4 show that the positioning accuracy of the Mate30 using the improved PPP project is obviously improved compare with the other two projects.

**Figure 11:**
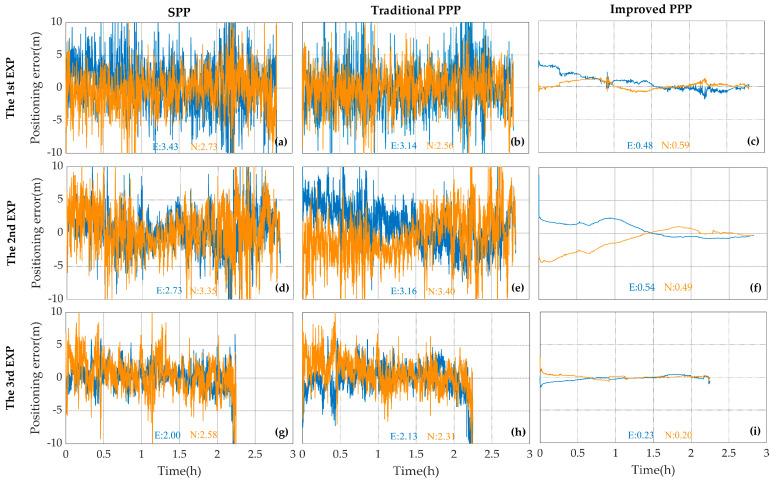
The E (blue) and N (orange) error components of SPP (**a**,**d**,**g**), the traditional PPP (**b**,**e**,**h**) and the improved PPP (**c**,**f**,**i**) results with the Mate30 during the first (**a**–**c**), the second (**d**–**f**) and the third (**g**–**i**) observing periods. The standard deviations of positioning errors are shown with the corresponding color in meters.

The time series of horizontal positioning errors are shown in Figure 11. When the improved PPP model is employed, the positioning errors in E and N directions during all of the three time periods can converge to less than 1 m and stay relatively stable. With the SPP and traditional PPP model, the positioning errors in E and N directions exceed 2 m, and the positioning results are unstable. In general, the performance of SPP is highly related to the data quality of code pseudorange. The positioning accuracies with the traditional PPP and SPP models are at the same level, although precise orbit and clock products are adopted in the PPP model. The poor performance of the traditional PPP in the above experiments could be explained from two aspects. On one hand, the quality of the code measurements collected by the Mate30 is poor. One the other hand, frequent loss of lock for the satellites make it difficult for the Kalman filter to work with the smartphones. Compared with the traditional PPP model, the improved PPP model is able to smooth code pseudorange with Doppler and to detect cycle slip more effectively, so the positioning accuracy is significantly improved.

## 5. Conclusions and Discussion

In this paper, the GNSS raw observations of two smartphones are analyzed through synchronous observations with a geodetic receiver. To study the positioning performances with the smartphones, experiments are conducted with a V20 smartphone and a Mate30 smartphone, as well as a Trimble R8 receiver. With the ionosphere-constrained single-frequency PPP strategy, an improvement in smartphone positioning is achieved. The horizontal positioning accuracy better than 1 m can be reached with the Mate30 smartphone.

Compared with a Trimble R8 geodetic receiver and a V20 smartphone, the GNSS performance of the Mate30 is evaluated. Firstly, there is little difference between the number of observable satellites in the first frequency of the Mate30 and the Trimble R8 receiver. The number of observable satellites with dual-frequency measurements available of the Mate30 and the V20 is not enough for dual-frequency PPP. The satellites lock loss of the Mate30 and the V20 is more frequent than the Trimble R8. The satellite tracking of smartphones is related to its chip. Secondly, the carrier-to-noise ratio of the smartphones is worse than that of the Trimble R8. And the carrier-to-noise ratio of the Mate30 is better than that of the V20. The carrier-to-noise ratio of smartphones has no significant relationship with elevation angle of satellites. Meanwhile, the multipath effect of smartphones is more serious than the Trimble R8. The standard deviations of code multipath errors of the Mate30 and the V20 are about ten times that of the Trimble R8. The performances of the smartphones against multipath effects of are worse than that of the geodetic receiver.

Since the number of navigation satellite with dual-frequency signals observed by smartphones is not enough for dual-frequency PPP, ionosphere-constrained single-frequency PPP model is used for smartphones. In this study, the standard deviations of horizontal positioning errors with the Mate30 are less than 1 m and the Mate30 achieves a relatively stable positioning result. Comparing the different positioning projects, we believe that there are two main reasons for the poor performance using the traditional PPP project of smartphones. For the current smartphones, the data qualities of the code measurements are relatively poor, and the losses of lock for satellites occur frequently. The ionosphere-constrained single-frequency PPP model along with the cycle slip detection method proposed in this work, to a certain extent, circumvents the above disadvantages and has considerable applicability on the smartphones.

The GNSS chip and antenna performance of smartphones is worse than that of geodetic receivers because the design of smartphones needs to consider the beauty and portability. This leads to frequent satellite lock loss, poor code pseudorange quality and serious multipath effects. Therefore, improving hardware quality and algorithm research are effective methods to improve smartphones positioning accuracy. Although the dual-frequency PPP model is unrealistic for smartphones at present due to their incompetence in collecting dual-frequency measurements, it is expected that the performance of PPP with smartphones will be significantly improved in the future with both the rapid development of BDS and the iterative upgrades of smartphones. 

## Figures and Tables

**Table 1:** Performance features of the Mate30, the V20 smartphones and Trimble R8 geodetic receiver.

Devices	Android Version	GNSS Supported ^1^	Code	Carrier Phase
Mate30	10	G (L1+L5), R (G1), E (E1+E5A), C (B1), J (L1+L5)	Yes	Yes
V20	10	G (L1+L5), R (G1), E (E1+E5A), C (B1), J (L1+L5)	Yes	Yes
Trimble R8	\	G (L1+L2), R (G1+G2), E (E1+E5A), C (B1+B2), J (L1+L2)	Yes	Yes

^1^ G: GPS, R: GLONASS, E: Galileo, C: BDS, J: QZSS. The definition of frequency bands is described in RINEX 3.03 format [28].

**Table 2:** The detailed settings of the PPP with smartphone.

Setting Items	Details
Observations	Single-frequency pseudorange and carrier phase
Satellite systems	GPS and BDS
Satellite orbit and clock	Precise orbit and clock product
Ionospheric delay	Estimated as a parameter
Tropospheric delay	The hydrostatic delay is corrected by the Saastamoinen model and the wet delay is estimated as a parameter
Effects of relativity and earth rotation	Earth rotation files
Weighting method	Satellite elevation angle
Integer ambiguities of carrier phase	Estimating float solution
Cutoff satellite elevation angle	10°
Parameters estimation method	Standard static Kalman filter

**Table 3:** The differences of detailed settings of positioning projects, where improved PPP employs ionosphere-constrained single-frequency PPP model described in Section 2. The other settings of the three projects employed the setting proposed in Table 2.

Options	Processing Strategies		
	SPP	Traditional PPP	Improved PPP
Code preprocessing	No	Gross error elimination [22]	The preprocessing strategy proposed in Section 2.2
Cycle slip detection	No	The strategy based on satellite lock out [22]	The strategy proposed in Section 2.3
Filtering processing	No	Yes	Yes

**Table 4:** The standard deviations of positioning errors in east (E) and north (N) directions.

Time Periods	Standard Deviations of Positioning Errors (m)					
	SPP		Traditional PPP		Improved PPP	
	E	N	E	N	E	N
1st	3.43	2.73	3.14	2.56	0.48	0.59
2nd	2.73	3.35	3.16	3.40	0.54	0.49
3rd	2.00	2.58	2.13	2.31	0.23	0.20

## References

[1] Li X., Ge M., Dai X., Ren X., Fritsche M., Wickert J., Schuh H. (2015). Accuracy and reliability of multi-GNSS real-time precise positioning: GPS, GLONASS, BeiDou, and Galileo. J. Geod..

[2] Yang Q., Li S. (2019). Analysis of the impact of open GNSS original measurement on the positioning accuracy of Android platform. J. Navig. Position..

[3] Google GPS Measurement Tools. https://github.com/google/gps-measurement-tools/tree/master/GNSSLogger.

[4] Logging of GNSS Raw Data on Android. http://www.geopp.de/logging-of-gnss-rawdata-on-android/.

[5] NSL Launches a New Free Android App as Part of FLAMINGO: Discover rinexON [Z/OL]. [2019–04–17]. https://www.flamingognss.com/rinexon.

[6] Gim J., Park K. (2017). Comparison of Positioning Accuracy Using the Pseudorange from Android GPS Raw Measurements. J. Korea Narvig Inst..

[7] Realini E., Caldera S., Pertusini L., Daniele S. (2017). Precise GNSS Positioning Using Smart Devices. Sensors.

[8] Zhang X., Tao X., Zhu F., Wang F. (2018). Quality assessment of GNSS observations from an Android N smartphone and positioning performance analysis using time-differenced filtering approach. GPS Solut..

[9] Hakansson M. (2019). Characterization of GNSS observations from a Nexus 9 Android tablet. GPS Solut..

[10] Li J., Bi J., Li D., Zhou W., Zhu S. (2017). Research on real-time BDS+GPS dual system wide area differential positioning technology under Android platform. Surv. Mapp. Bull..

[11] Specht C., Dabrowski P.S., Pawelski J., Specht M., Szot T. (2019). Comparative analysis of positioning accuracy of GNSS receivers of Samsung Galaxy smartphones in marine dynamic measurements. Adv. Space Res..

[12] Dabove P., Pietra C.D. (2019). Towards high accuracy GNSS real-time positioning with smartphones. Adv. Space Res..

[13] Wanninger L., Hesselbarth A. (2020). GNSS code and carrier phase observations of a Huawei P30 smartphone: Quality assessment and centimeter-accurate positioning. GPS Solut..

[14] World’s First Dual-Frequency GNSS Smartphone Hits the Market. https://www.gsa.europa.eu/newsroom/news/world-s-first-dual-frequency-gnss-smartphone-hits-market.

[15] Zhao S., MI J., Xu Y., Zhao Z. (2020). Data quality and positioning accuracy analysis of dual-frequency smart phone GNSS [J/OL]. Surv. Mapp. Sci..

[16] Chen B., Gao C., Liu Y., Lu Y. (2019). Quality analysis on raw GNSS measurements of Android mobile terminals. J. Navig. Position..

[17] Chen B., Gao C., Liu Y., Sun P. (2019). Real-time precise point positioning with a Xiaomi MI-8 android smartphone. Sensors.

[18] Shi X. (2019). Continuous Smoothing Positioning Algorithm Based on GNSS Observations of Smartphones. Master’s Thesis.

[19] Wu Q., Sun M., Zhou C., Zhang P. (2019). Precise Point Positioning Using Dual-Frequency GNSS Observations on Smartphone. Sensors.

[20] Aihetamu Y., Huang Z., Wang Y. (2017). Discussion on GPS Single-frequency PPP Ionosphere Delay Correction Model. J. Gansu Sci..

[21] Shi C., Gu S., Lou Y., Ge M. (2012). An Improved Approach to Model Ionospheric Delays for Single-frequency Precise Point Positioning. Adv. Space Res..

[22] Zhou F., Dong D.N., Li W.W., Jiang X.Y., Wickert J., Schuh H. (2018). GAMP: An open-source software of multi-GNSS precise point positioning using undifferenced and uncombined observations. GPS Solut..

[23] Kouba J. A Guide to Using International GNSS Service (IGS) Products. http://iacc.igs.org/UsingIGS-ProductsVer21.pdf.

[24] Xiao Q., Gu S., MI J., Chen B., Chen C. (2020). Doppler smoothed pseudorange single point positioning accuracy analysis for smartphones. Surv. Mapp. Sci..

[25] Zhao J.J., Hernandez-Pajares M., Li Z.S., Wang L., Yuan H. (2020). High-rate Doppler-aided cycle slip detection and repair method for low-cost single-frequency receivers. GPS Solut..

[26] Qian C., Liu H., Zhang M., Shu B., Xu L.W., Zhang R.F. (2016). A Geometry-Based Cycle Slip Detection and Repair Method with Time-Differenced Carrier Phase (TDCP) for a Single Frequency Global Position System (GPS) plus BeiDou Navigation Satellite System (BDS) Receiver. Sensors.

[27] Zangeneh-Nejad F., Amiri-Simkooei A.R., Sharifi M.A., Asgari J. (2017). Cycle slip detection and repair of undifferenced single-frequency GPS carrier phase observations. GPS Solut..

[28] International GNSS Service (IGS), RINEX Working Group and Radio Technical Commission for Maritime Services Special Committee 104 (RTCM-SC104) The Receiver Independent Exchange Format. ftp://igs.org/pub/data/format/rinex303.pdf.

[29] Wu D., Wang L., Zhang Q. (2015). Implementation and verification analysis of GNSS data quality evaluation software. J. Surv. Mapp. Sci. Technol..

[30] Bilich A. (2008). Mapping the GPS multipath environment using the signal-to-noise ratio. Radio Sci..

[31] Axelrad P., Comp C., Macdoran P.F. (1996). SNR-Based multipath error correction for GPS differential phase. IEEE Trans. Areospace Electron. Syst..

[32] Zhang X., Ding L. (2013). Quality Analysis of the Second Generation Compass Observables and Stochastic Model Refining. Geomat. Inf. Sci. Wuhan Univ..

